# Effects of straw and plastic film mulching on microbial functional genes involved in soil nitrogen cycling

**DOI:** 10.3389/fmicb.2023.1205088

**Published:** 2023-07-11

**Authors:** Ying Dou, Mengmeng Wen, Caidi Yang, Fazhu Zhao, Chengjie Ren, Nannan Zhang, Yinyan Liang, Jun Wang

**Affiliations:** ^1^Shaanxi Key Laboratory of Earth Surface System and Environmental Carrying Capacity, College of Urban and Environmental Sciences, Northwest University, Xi’an, China; ^2^College of Agronomy, Northwest A&F University, Yangling, China; ^3^State Key Laboratory of Soil Erosion and Dryland Farming on the Loess Plateau, Institute of Soil and Water Conservation, Chinese Academy of Sciences and Ministry of Water Resources, Yangling, China

**Keywords:** straw mulching, plastic film mulching, nitrogen cycling, microbial functional genes, microbial community

## Abstract

**Introduction:**

Microorganisms regulate soil nitrogen (N) cycling in cropping systems. However, how soil microbial functional genes involved in soil N cycling respond to mulching practices is not well known.

**Methods:**

We collected soil samples from a spring maize field mulched with crop straw (SM) and plastic film (FM) for 10-year and with no mulching (CK) in the Loess Plateau. Microbial functional genes involved in soil N cycling were quantified using metagenomic sequencing. We collected soil samples from a spring maize field mulched with crop straw (SM) and plastic film (FM) for 10-year and with no mulching (CK) in the Loess Plateau. Microbial functional genes involved in soil N cycling were quantified using metagenomic sequencing.

**Results:**

Compared to that in CK, the total abundance of genes involved in soil N cycling increased in SM but had no significant changes in FM. Specifically, SM increased the abundances of functional genes that involved in dissimilatory nitrate reduction to ammonium (*nirB*, *napA*, and *nrfA*), while FM decreased the abundances of functional genes that involved in ammonification (*ureC* and *ureA*) in comparison with CK. Other genes involved in assimilatory nitrate reduction, denitrification, and ammonia assimilation, however, were not significantly changed with mulching practices. The *nirB* and *napA* were derived from *Proteobacteria* (mainly *Sorangium*), and the *ureC* was derived from *Actinobacteria* (mainly *Streptomyces*). Mental test showed that the abundance of functional genes that involved in dissimilatory nitrate reduction was positively correlated with the contents of soil microbial biomass N, potential N mineralization, particulate organic N, and C fractions, while ammonification related gene abundance was positively correlated with soil pH, microbial biomass C and N, and mineral N contents.

**Discussion:**

Overall, this study showed that SM could improve soil N availability and promote the soil N cycling by increasing the abundance of functional genes that involved in DNRA, while FM reduced the abundance of functional genes that involved in ammonification and inhibited soil N cycling.

## Highlights

– Straw mulching increased all soil N fractions while plastic film mulching reduced MBN but increased mineral N.
– The total abundance of N cycling-related functional genes increased with straw mulching but did not change with film mulching.
– Straw mulching increased the abundances of functional genes in DNRA (*nirB*, *napA*, and *nrfA*).
– Plastic film mulching decreased the abundances of functional genes in ammonification (*ureC* and *ureA*).
– The abundances of N cycling-related functional genes were associated with soil pH and C and N fractions.

## 1. Introduction

Soil nitrogen (N) cycling is one of the most important processes in agroecosystems, which not only affects soil fertility and crop productivity, but also affects the sustainable development of agriculture (Zhang et al., 2013; Wu et al., 2021). The N cycling processes in soil, including nitrogen fixation, ammonification, nitrification, denitrification, dissimilatory nitrate reduction to ammonium (DNRA), and assimilatory nitrate reduction (ANR), are mainly driven by soil microorganisms (Kuypers et al., 2018). The dynamic core formed by microbial community structure and function drives the decomposition and mineralization of plant residues, thus affecting soil N cycling (Wang et al., 2020). Previous studies have shown that the *amoA* regulates the utilization of inorganic N and the production of N_2_O by regulating the first step in nitrification (Dai et al., 2020; Li et al., 2020). In denitrifying process, the *napA* and *narG* participate in the reduction of NO_3_^−^ to NO_2_^−^, the *nirK* and *nirS* convert NO_2_^−^ to NO, and *norB* and *nosZ* mediate the conversion of NO to N_2_ (Zumft, 1997; An et al., 2022). In addition, the *gdh* and *ureC* are key genes in the N mineralization (Li et al., 2020; Kushwaha et al., 2021; Yu et al., 2022). Therefore, understanding and analyzing the microbial functional genes coding for N cycling is crucial for regulating specific microbes.

Surface mulching with crop straw or plastic film has been widely adopted to improve water use efficiency and crop production, especially in dryland cropping systems (Hu Y. J. et al., 2021). Compared to no mulching, straw mulching as a low-cost and off-the-shelf practice can provide long-term benefits by improving soil properties, nutrient cycling and enzyme activity (Luo et al., 2021). Straw mulching can increase soil organic matter, improve the availability of substrate, provide a good living environment for microorganisms, improve microbial activity, and then affect the N cycling (Vassilev et al., 2010; Cui et al., 2017; Yang et al., 2023). As another important measure in dryland cropping systems, plastic film mulching can stimulate soil microbial activity by improving soil hydrothermal conditions (Liu et al., 2015; Lee et al., 2019), which will affect soil N cycling (Hu Y. J. et al., 2021). The findings of previous studies have reported that straw mulching could increase the abundance and activity of soil fungi, while the effects on bacterial abundance and activity were inconsistent (Dong et al., 2017; Fu et al., 2019). Furthermore, straw could increase soil available C and N contents, thus promoting the activities of nitrifiers and denitrifiers (Huang R. et al., 2019). For FM, some studies found that it significantly increased the diversity and richness fungi but decreased those of bacteria (Fu et al., 2019; Huang F. Y. et al., 2019). Other researchers also shown that soil N mineralization rate would be enhanced with film mulching due to the increased microbial activities (Hai et al., 2015; Dong et al., 2018; Luo et al., 2019). Although the responses of soil microbial diversity and community structure to mulching practices have been well reported, how straw mulching and film mulching affect the functional genes involved in soil N cycling remains unclear.

Based on a 10-year field experiment in the Loess Plateau of China, the effects of straw mulching and film mulching on microbial functional genes that involved in soil N cycling were explored in comparison with no mulching using metagenomic sequencing. We hypothesized that surface mulching with crop straw or film mulching will increase the abundances of functional genes in N cycling by increasing substrate supply or altering soil environmental conditions. We aimed to: (1) clarify how straw mulching and film mulching affect the abundances of microbial functional genes that involved in different processes of soil N cycling; (2) determine the main factors affecting microbial functional genes that involved in N cycling under different mulching practices.

## 2. Materials and methods

### 2.1. Site description

A field experiment was conducted in 2008 at the Changwu Agro-Ecological Research Station in the Loess Plateau of China (107°45′ E, 35°12′ N; 1,200 m elevation). The station has a continental monsoon climate with a mean air temperature of 9.1°C and a mean annual precipitation of 584 mm. The precipitation mainly occurred in summer fallow period from July to September. The soil is a Heilutu silt loam (Calcarid Regosol according to the FAO classification system), with the contents of sand, silt, and clay of 45, 656, and 309 g kg^−1^, respectively. At the beginning of the experiment in September 2008, the soil has a soil organic carbon (SOC) of 10.5 g kg^−1^, total N (STN) of 0.80 g kg^−1^, total phosphorus (TP) of 0.81 g kg^−1^, and available phosphorus (AP) of 5.34 mg kg^−1^ at 0–20 cm.

### 2.2. Experimental design

Three treatments, as straw mulching (SM), plastic film mulching (FM), and no mulching (CK), were arranged in a completely randomized block design with three replications in a spring maize (*Zea mays* L.) field. Each treatment has three plots, and the plot has a length of 10.3 m and a width of 6.5 m. Spring maize was planted in mid to late April and harvested in early October each year, after which the test plots are left idle. Crop straw or mulch were removed away by hand before sowing. The basal fertilization were carried out with urea (*N* ≥ 46.6%) and superphosphate (total P_2_O_5_ ≥ 43%) at rates of 120 kg N ha^−1^ and 60 kg P ha^−1^ at sowing, respectively. In SM, maize straw was placed on the soil surface at planting immediately after sowing. In FM, 1 mm-thick plastic film mulch was used covering the soil surface with edges covered by soil particles and then maize was sown using a hill planter. More detailed descriptions could be found in our previous report by Wang et al. (2018).

### 2.3. Soil collection and laboratory analyses

Soil sampling was conducted after 10 years in early October 2018. In each plot, five cores were randomly collected at 0–10 cm using a hand probe (with a diameter of 2.5 cm inside) and then composited. Part of the soil samples were frozen at −80°C immediately for DNA extraction and metagenomic sequencing. The remaining samples were air-dried and screened to 2 mm for laboratory analyses. Soil pH was determined in a soil: water mixture at a 1:2.5 (w/v) ratio using a glass electrode meter (Mettler Toledo FE28- Standard) (Zhang et al., 2016; Fu et al., 2019). The content of SOC was measured using the H_2_SO_4_-K_2_Cr_2_O_7_ method (Nelson and Sommers, 1996). The STN content was determined using the Kjeldahl method (Purcell and King, 1996). The microbial biomass C and N (MBC and MBN) were analyzed using the chloroform fumigation extraction method (Vance et al., 1987; Wu et al., 1990). The potential C and N mineralization (PCM and PNM) were determined using the closed culture method (Jenkinson and Powlson, 1976; Goyal et al., 1999). To determine soil ammonium (NH_4_^+^-N) and nitrate (NO_3_^−^-N) concentrations, samples were extracted with a 2 mol L^−1^ KCl solution, and the extracts were analyzed using a Dionex ICS 1500 ion chromatograph (Dionex Co., Sunnyvale, CA) (Zhang et al., 2014). Soil property data were listed in Supplementary Table S1.

### 2.4. DNA extraction, sequencing, and data processing

According to the manufacturer’s instructions, soil DNA was extracted from 0.5 g of fresh soil samples using the FastDNA Soil Rotation kit (MP Biomedicals, Cleveland, United States) (Ren et al., 2016; Wang et al., 2022). The quality and purity of DNA extract were evaluated using the Nanodrop 2000 spectrophotometer. Each soil sample was repeated for 3 times to obtain enough DNA for shotgun metagenomic sequencing. The metagenome was sequenced using an Illumina HiSeq 2000 platform (Personal, Shanghai, China) to generate 150 bp paired-end reads at a high sequencing depth. Reads that aligned to the human genome were removed, and the lengths of the remaining reads were trimmed using Sickle. Data of soil DNA sequences are accessible on the website of National Center for Biotechnology Information,^1^ with the accession number of PRJNA876629.

### 2.5. Metagenomic analysis

Raw sequencing readings were filtered to improve the reliability and quality of subsequent analyses (Zhang et al., 2017). The ambiguous bases, adapter sequences, and reads that were less than 50 bp in length were removed using the fastp^2^ on the free online platform of Majorbio Cloud Platform^3^ (Chen et al., 2022). The resulting clean reads were assembled to contig using Megahit^4^ with the optimal k-mer parameter (Li et al., 2015). Then, contigs with sequences (length of more than 300 bp) were used to predict the open reading frame (ORFs) using MetaGeneMark^5^ (Zhu et al., 2010). All the above protein sequences into a non-redundant gene catalog were clustered in CD-HIT technique, with 90% protein similarity and 90% coverage (Fu et al., 2012). Reads after quality control were mapped to the non-redundant gene catalog with 95% identity using SOAPaligner,^6^ and evaluated gene abundance information in the corresponding samples (Li et al., 2008). We determined the trans per million values [TPM: (Reads Number/Gene Length) _Relative] × 100,0000 in gene abundance for each sample (Qin et al., 2012). On the basis of the NCBI NR database, the representative sequences of non-redundant gene catalog were annotated with using blastp as implemented in DIAMOND v0.9.19 with e-value cutoff of 1e^−5^ using Diamond^7^ for taxonomic assignments (Buchfink et al., 2015). According to the Kyoto Encyclopedia of Genes and Genomes (KEGG) database,^8^ the functional annotation and taxonomic assignment of the sequences obtained for each sample were performed, and then the functional genes involved in N cycling were selected. Based on previous studies (Nelson et al., 2016; Kuypers et al., 2018), the 28 microbial functional genes found in metagenome were defined into the following six subgroups: (1) ammonification, (2) nitrification, (3) denitrification, (4) dissimilatory nitrate reduction to ammonium (DNRA), (5) assimilatory nitrate reduction (ANR), and (6) ammonia assimilation. Details for each gene involved in soil N cycling and its function were summarized in Supplementary Table S2.

### 2.6. Statistical analysis

The differences of the abundances of microbial functional genes and soil properties among treatments were analyzed using the least significant difference (LSD) test by SPSS 25.0 software. The overall differences in microbial functional gene composition for soil N cycling among treatments were tested using analysis of similarities (ANOSIM) and principal coordinates analysis (PCoA) based on the Bray-Curtis distance. The relationships between the abundances of microbial functional gene groups that involved in soil N cycling and soil properties was determined using the Mantel test. Both ANOSIM, PCoA, and Mantel test were performed in R 4.1.2 software.

## 3. Results

### 3.1. Soil physicochemical properties

**Figure 1:**
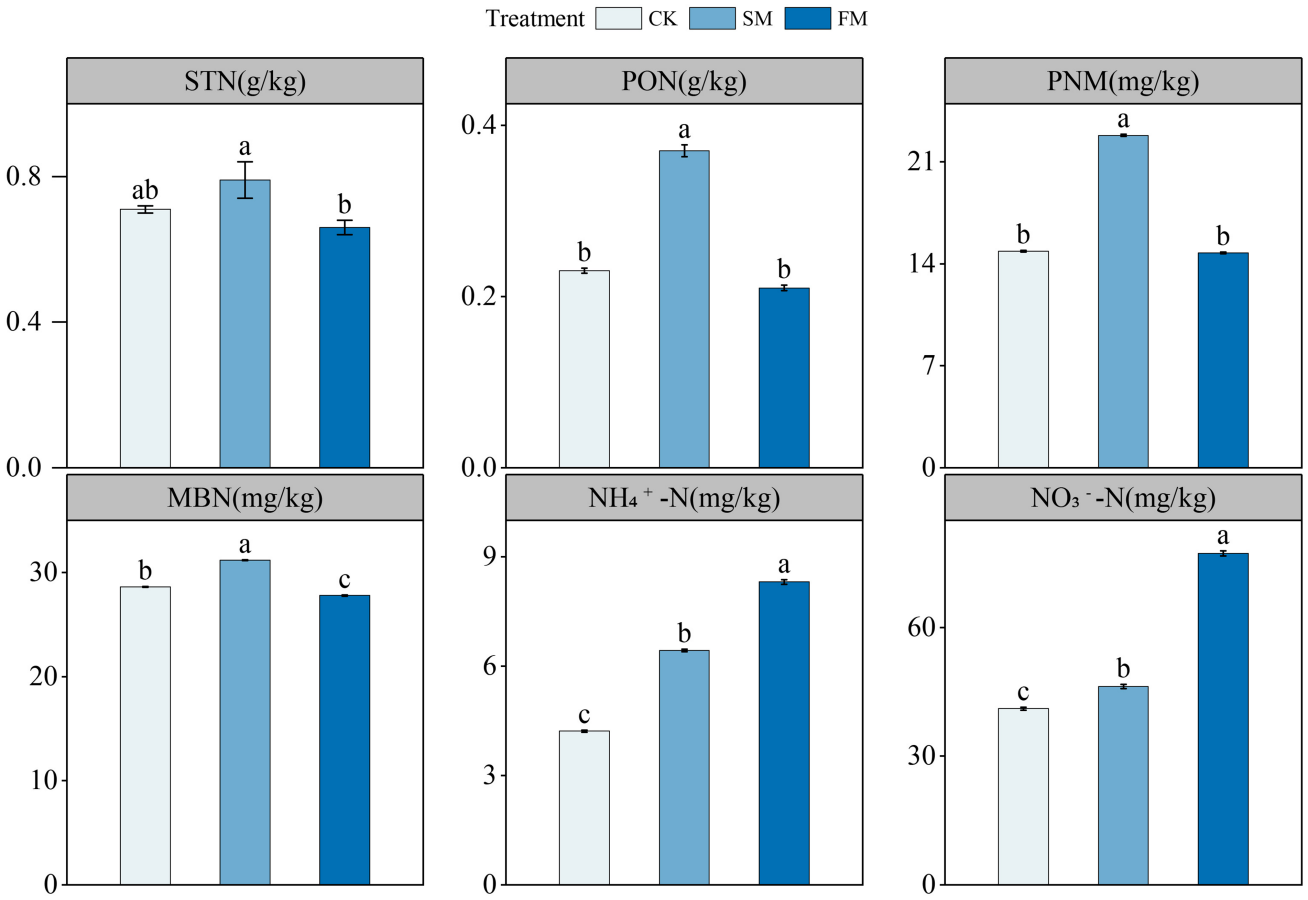
The contents of soil N fractions under different mulching. Values are means ± standard error (*n* = 3). Different letters indicate significant differences (*p* < 0.05) between the treatments. SM, straw mulching; FM, plastic film mulching; and CK, no mulching. MBN, microbial biomass N; PNM, potential N mineralization; PON, particulate organic N; STN, soil total N; NH_4_^+^-N, ammonium; NO_3_^−^-N, nitrate N.

The STN content was significantly higher in SM than in FM (Figure 1). Compared to those in CK, the contents of MBN, PNM, and PON significantly increased by 9.04, 53.5, and 61.76% (*p* < 0.05) in SM, respectively. No differences in the contents of PNM and PON in FM, but that of MBN significantly decreased by 2.85% (*p* < 0.05). The contents of NH_4_^+^-N and NO_3_^−^-N significantly increased by 52.35, 12.6, 96.98, and 88.18% (*p* < 0.05) in SM and FM, respectively.

The other relevant soil physicochemical properties were shown in Supplementary Table S1. The contents of SOC and MBC were significantly higher in SM than in CK and FM. Soil pH and the contents of SOC and MBC were significantly lower in FM than in CK. The ratios of C:N were not different among treatments.

### 3.2. Microbial functional genes related to soil N cycling

**Figure 2:**
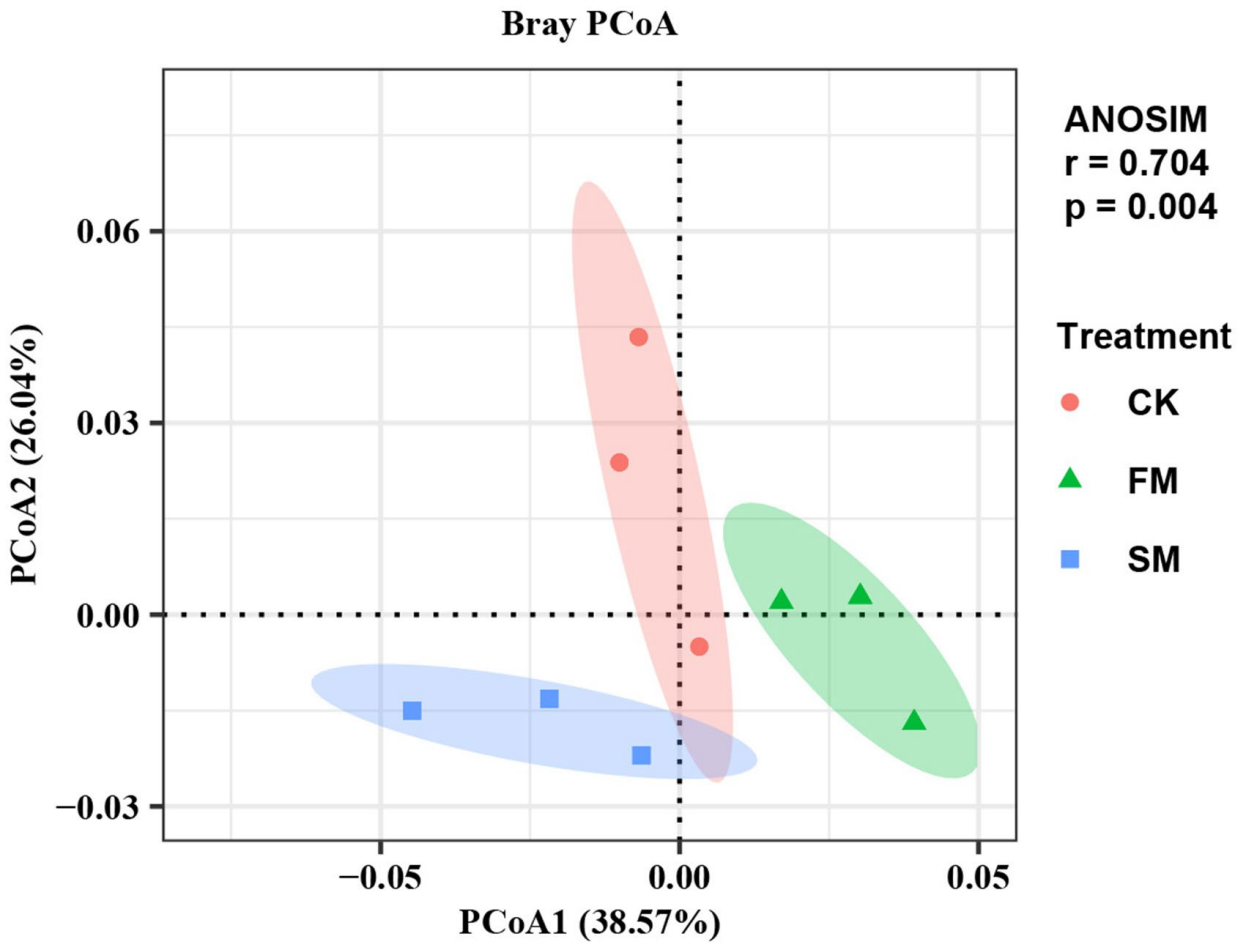
Principal coordinates analysis (PCoA) and analysis of similarities (ANOSIM) of microbial functional gene composition involved in soil N cycling under different mulching based on Bray-Curtis distances. SM, straw mulching; FM, plastic film mulching; and CK, no mulching.

The abundances of microbial functional genes that involved in soil N cycling varied with mulching practices according to ANOSIM and PCoA analysis (Figure 2). The total abundance of functional genes that involved in soil N cycling was higher in SM than in CK, but did not significant change in FM.

**Figure 3:**
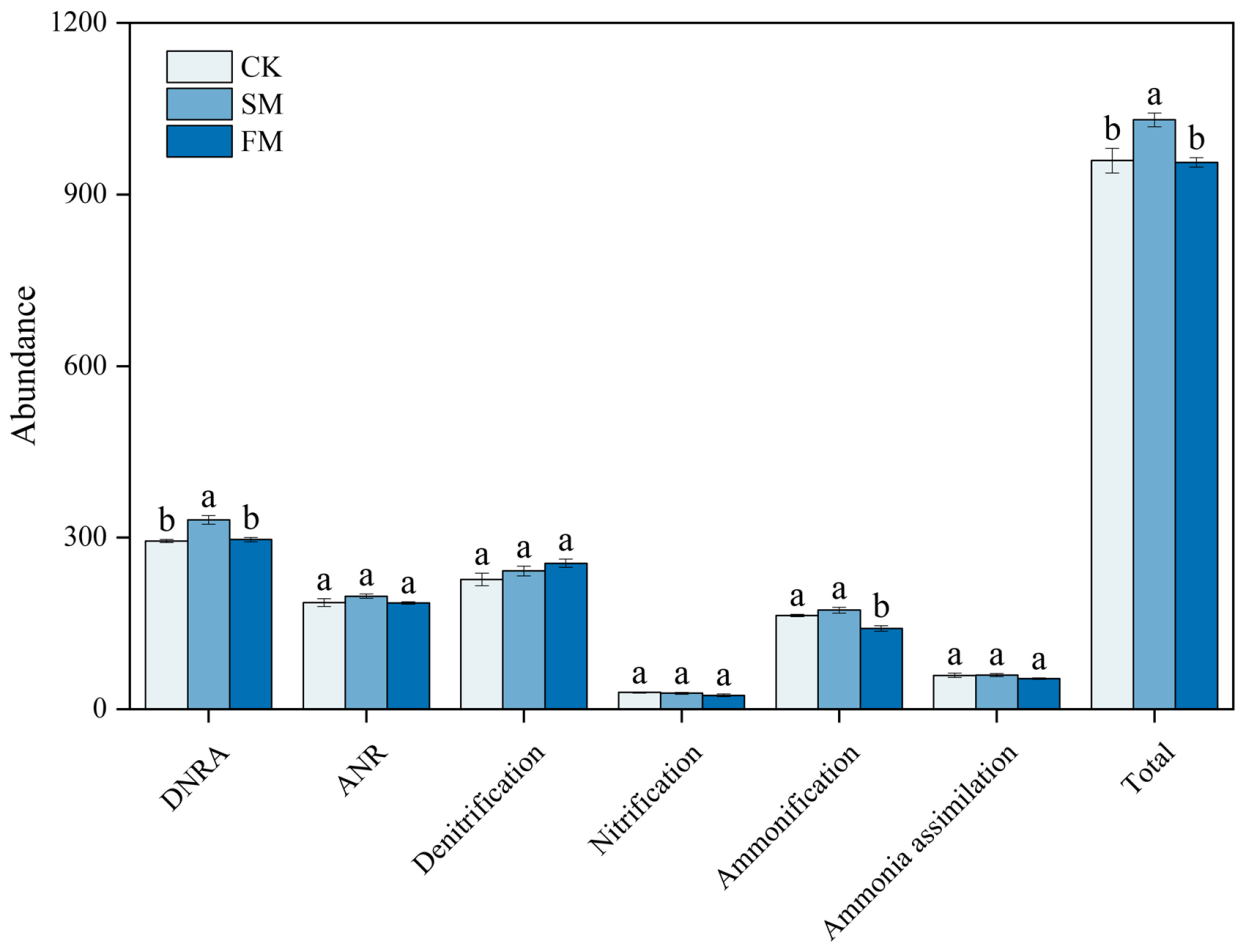
The total abundance of all functional genes involved in different N cycling processes under different mulching. SM, straw mulching; FM, plastic film mulching; and CK, no mulching. Different letters above the bar indicate significant differences among treatments (*p* < 0.05).

For N-cycling genes, the abundance was the highest in DNRA, followed by Denitrification, ANR, Ammonification, Ammonia assimilation and nitrification (Figure 3). Compared to those in CK, the total abundance of functional genes that involved in DNRA was significantly higher by 12.63% in SM and that in ammonification was significantly lower by 13.93% in FM (*p* < 0.05). The total abundances of functional genes that involved in nitrification, denitrification, ANR, and ammonia assimilation processes were not significant among treatments.

**Figure 4:**
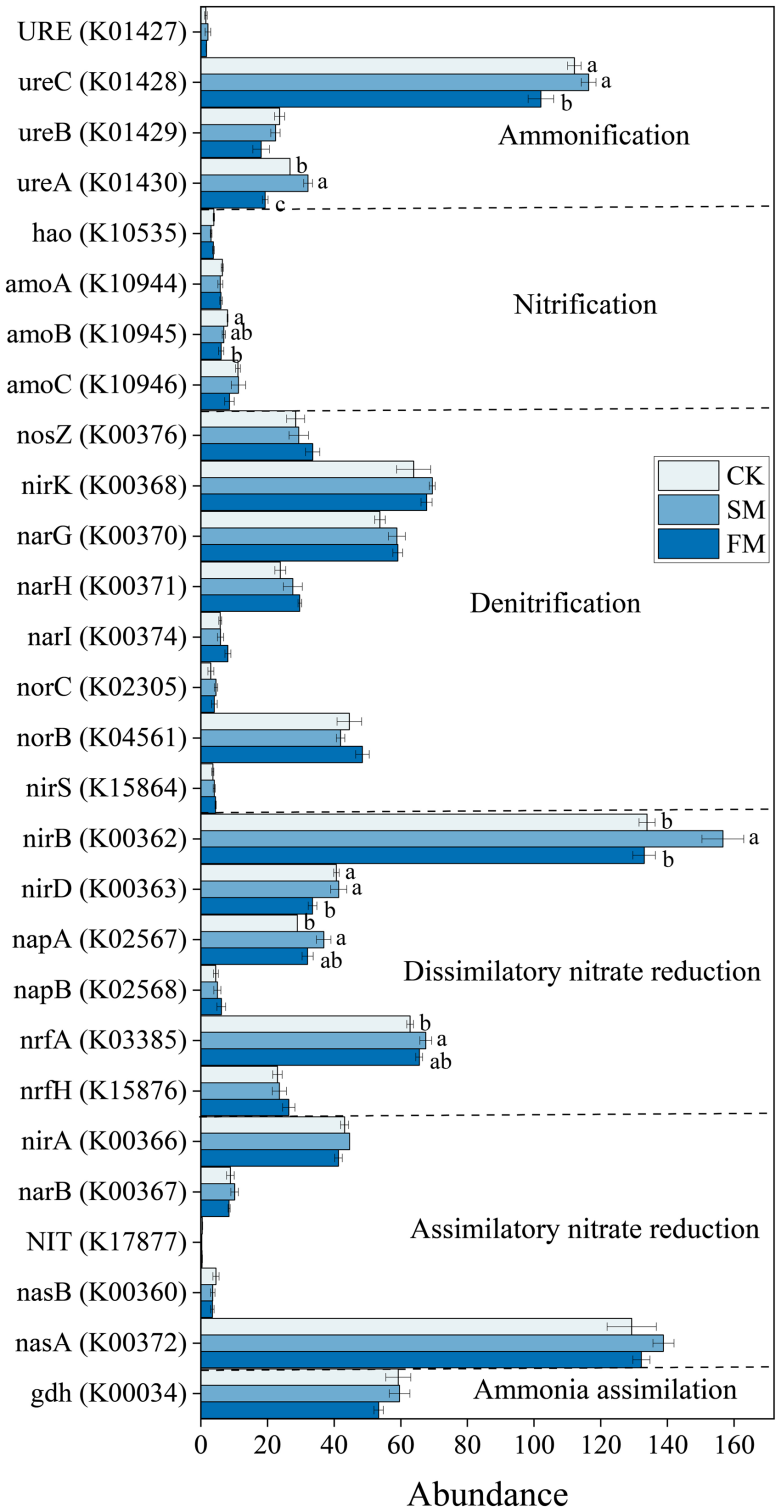
The abundance of functional genes involved in soil N cycling under different mulching. Values are means ± standard error (*n* = 3). Different letters indicate significant differences (*p* < 0.05) between the treatments. SM, straw mulching; FM, plastic film mulching; and CK, no mulching.

The gene abundances of *ureC*, *nirB*, and *nasA* were significantly higher than others. Specifically, the gene abundances of *nirB*, *napA*, and *nrfA* in DNRA and *ureA* in ammonification were significantly higher by 17.00, 27.46, 7.47, 20.36% (*p* < 0.05) in SM than in CK (Figure 4), respectively. However, the gene abundances of *nirD* in DNRA, *ureC*, *ureA* in ammonification, and *amoB* in nitrification were significantly lower by 17.63, 8.99, 27.84, and 23.90% (p < 0.05) in FM than in CK, respectively.

### 3.3. Taxonomic assignments of genes involved in soil N cycling

**Figure 5:**
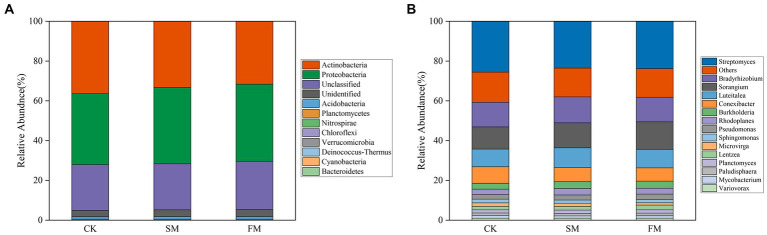
Relative abundances of bacterial involved in soil N cycling under different mulching for **(A)** phyla (relative abundance >0.1%); **(B)** genera (relative abundance >1%). SM, straw mulching; FM, plastic film mulching; and CK, no mulching.

The effects of SM and FM on the relative abundances of bacterial phyla and genera related to N-cycling related genes are shown in Figure 5. We detected a total 10 bacterial phyla containing N-cycling functional genes. Compared with that in CK, SM and FM significantly decreased the relative abundance of *Actinobacteria*, while significantly increased the relative abundance of *Proteobacteria* and *Verrucomicrobia* (*p* < 0.05). In addition, SM also significantly decreased the relative abundance of *Chloroflexi*, and FM significantly increased the abundances of *Bacteroidetes* and *Deinococcus-Thermus* (*p* < 0.05), when compared to CK. For the bacterial genera, SM and FM significantly decreased the relative abundance of *Conexibacter*, but significantly increased the relative abundance of *Pseudomonas* and *Sorangium* (*p* < 0.05). The relative abundance of *Burkholderia* and *Rhodoplanes* significantly increased only in SM, while the relative abundance of *Microvirga*, *Mycobacterium*, *Sphingomonas*, and *Streptomyces* significantly decreased only in FM (*p* < 0.05).

### 3.4. Linkages between soil N cycling functional genes and environmental parameters

**Figure 6:**
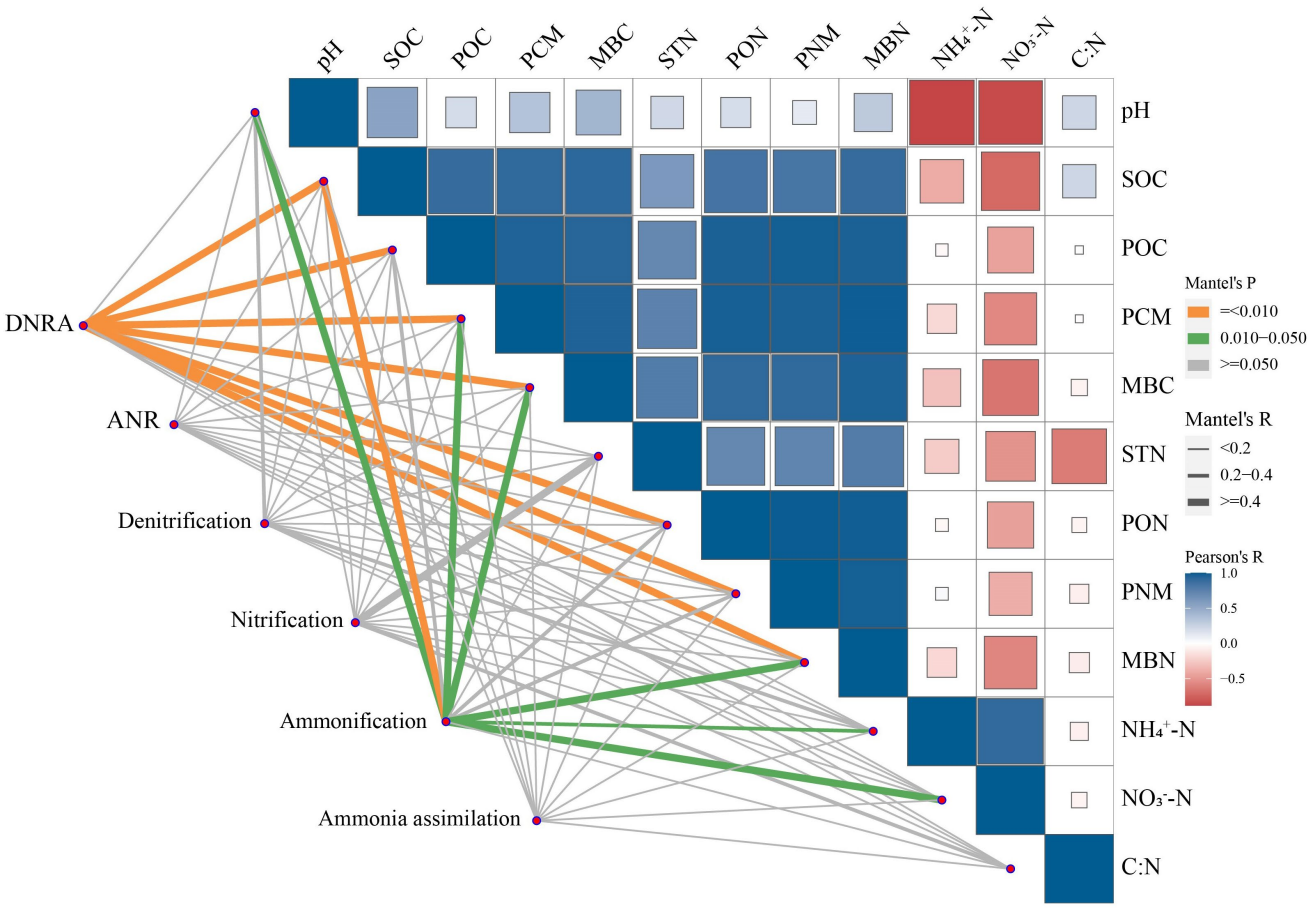
Correlations among soil properties and the abundance of microbial functional gene groups involved in N cycling in soil. Microbial functional gene groups were related to soil properties by Mantel test (green and orange line indicate significant at *p* < 0.05 and 0.01 levels, respectively). The correlations between soil properties are shown with the intensity of color (blue and red a positive and negative correlation, respectively).

According to the Mantel test, the abundance of functional genes that involved in DNRA was positively correlated with the contents of soil C and N fractions except for STN, NH_4_^+^-N, and NO_3_^−^-N (*p* < 0.01, Figure 6; Supplementary Table S3). Similarly, the abundance of functional genes that involved in ammonification exhibited positive correlations with soil pH and the contents of SOC, MBC, PCM, MBN, NH_4_^+^-N, and NO_3_^−^-N (*p* < 0.05). No significant correlations were found between the abundances of functional genes that involved in other processes and soil pH, C and N fractions, and C: N.

## 4. Discussion

### 4.1. Effects of mulching practices on soil N fractions

The larger N fractions in SM than in CK (Figure 1) should be due to the increased N input through crop straw decomposition (Gu et al., 2016; Fu et al., 2019; Li et al., 2021). The decreases of STN and MBN in FM (Figure 1) was possibly because of stimulated mineralization of soil organic matter due to the enhanced soil hydrothermal conditions under film mulching (Liu et al., 2009). Notably, we found that the contents of NH_4_^+^-N and NO_3_^−^-N were higher in SM and FM than CK (Figure 1). The mineralization of soil organic N is mainly controlled by soil environmental conditions and microbial activities (Pramanik et al., 2017; Waldrop et al., 2017). For SM, long-term mulching has a good effect on insulation and can also increase soil organic matter content, which may create a favorable environment for soil microbial community involved in N transformation, thus enhancing the mineralization of organic N, which lead to the increase in soil mineral N (Zhang et al., 2009; Yang et al., 2023). For FM, the increased N hydrolysis and reduced ammonia volatilization could reduce the loss consumption of N, thus resulting in higher soil NH_4_^+^-N and NO_3_^−^-N contents (Ding et al., 2021).

### 4.2. Effects of mulching practices on microbial functional genes that involved in soil N cycling

The total abundance of functional genes that involved in soil N cycling increased in SM but not significant in FM (Figure 3), indicating a differential response of functional genes involved in soil N cycling to mulching practices.

The increased genes involved in soil N cycling in SM was mainly from those related to DNRA (Figure 3). The process of DNRA was strongly controlled by the supplies of soil C and N substrates, and high availabilities of C and N would promote the occurrence of DNRA (Chen et al., 2015; Cheng et al., 2022). Zhang et al. (2019) reported that the abundance of genes that involved in the DNRA increased with the increasing N input through fertilization. In this study, the abundance of functional genes that involved in DNRA was positively correlated with C and organic N fractions except for STN and mineral N (Figure 6; Supplementary Table S3), indicating that the level of soil C rather than N content is the main factor affecting DNRA under straw mulching. The increased soil C contents due to straw mulching (Supplementary Table S1) could provide electrons through fermentation or respiration and convert NO_3_^−^ into NH_4_^+^ in the soil, thus providing energy for DNRA bacteria and increasing the gene abundances in this process (Yoon et al., 2015; Van Den Berg et al., 2016). The stimulation of functional genes that involved in DNRA process could reduce the volatilization of gaseous N and the risk of nitrate leaching in denitrification process by converting the NO_3_^−^ into NH_4_^+^, which could facilitate the retention of N in agricultural soil (Pan et al., 2020; Pandey et al., 2020). At the gene level, SM mainly increased the abundances of *nirB*, *napA*, and *nrfA* (Figure 4). Similarly, Bai et al. (2020) also reported that the abundance of *nrfA* increased after straw incorporation. The *napA* encoding the dissimilatory nitrate reductase that catalyzed the reduction of NO_3_^−^ to NO_2_^−^, the *nrfA* and *nirB* encoding the nitrite reductase enzymes that catalyzed the reduction of NO_2_^−^ to NH_4_^+^, and these gene are frequently used as markers for DNRA process (Mohan et al., 2004; Pandey et al., 2020; An et al., 2022). Previous studies have shown that the gene abundances of *napA* and *nrfA* were positively correlated with soil C (Levy-Booth et al., 2014; Shi et al., 2020). In SM, the supply of high C substrate increased heterotrophic soil respiration, reduced the soil redox potential, and promoted the occurrence of DNRA thus increased the abundance of these genes (Chen et al., 2015; Putz et al., 2018; Kasak et al., 2021).

Although film mulching did not affect the total abundance of genes involved in soil N cycling significantly, it decreased the abundance of genes involved in ammonification (Figure 3). The process of ammonification was driven by soil pH and N substrates, and low soil pH and soil substrate may limit the expression of ammonification genes (Booth et al., 2005; Hu Y. Y. et al., 2021). In this study, the abundance of ammonification-related genes was also associated with the changes in soil pH and the contents of SOC, MBC, PCM, MBN, NH_4_^+^-N, and NO_3_^−^-N (Figure 6; Supplementary Table S3). The lower pH in FM than in CK (Supplementary Table S1) may have limited the growth and activity of microorganisms, inhibited enzyme activities during ammonification, and thus reduced the abundance of ammonification-related genes (Vitousek et al., 1997; Fisher et al., 2017). Also, the decreased the contents of SOC, MBC, and MBN under film mulching (Supplementary Table S1) reduced the substrates and energies for microorganism growth and basal metabolism, thus inhibiting the functional activity of microorganisms (Taylor et al., 2002; Tu et al., 2006; Jankowski et al., 2014). At the gene level, FM mainly decreased the abundances of *ureC* and *ureA* (Figure 4). However, Zhang et al. (2019) found that the *ureC* was enhanced by the increased soil exchangeable NH_4_^+^-N content with urea fertilization. This inconsistency may be attributed to the large increase of mineral nitrogen in FM (Figure 1), which might lead to the formation of refractory humus compounds, and correspondingly reduce the efficiency of extracellular microbial enzymes and inhibit the growth of microorganisms (Waldrop and Zak, 2006; Chen et al., 2016).

### 4.3. Effects of mulching practices on soil dominant microorganism with N cycling genes

*Actinobacteria* and *Proteobacteria* were the dominant phyla for soil (Figure 5). In our study, the relative abundance of *Actinobacteria* decreased, and *Proteobacteria* was increased both in SM and FM. This change may be because *Proteobacteria* belong to the copiotrophic groups, which have fast-growing rates and are more likely to increase under nutrient-rich conditions, while *Actinobacteria* belonging to the oligotrophic groups with a slower growth rate, would likely decline (Fierer et al., 2007; Zhang et al., 2019). We also performed the taxonomic assignments of key functional genes at the general level (Supplementary Figure S1). The results showed that *ureA* and *ureC* are mainly harbored in *Streptomyces*, *nirB* and *napA* are harbored in *Sorangium*. For SM, the relative abundance of *Sorangium* was increased, which may stimulate the expression of DNRA-related genes. For FM, the relative abundance of *Streptomyces* was decreased, thus may have limited the expression of these two genes that involved in ammonification. Moreover, Zhao et al. (2020) also found that genes involved in ammonification (*ureC*) were mainly derived from *Streptomyces*, which reinforces our conclusion.

## 5. Conclusion

The functional gene that involved in soil N cycling responded differentially to mulching practices, which is partly consistent with our hypothesis. The total abundance of functional genes that involved in soil N cycling increased with straw mulching but not significant with film mulching. Specifically, straw mulching increased the abundance of functional genes related to DNRA (*nirB*, *napA*, and *nrfA*), while film mulching decreased the abundances of those associated with ammonification (*ureC* and *ureA*). The functional genes related to assimilation nitrate reduction, denitrification, and ammonia assimilation were not affected by mulching practices. *Actinobacteria* (mainly *Streptomyces*) and *Proteobacteria* (mainly *Sorangium*) were the dominant phyla harboring functional genes that involved in soil N cycling. Soil pH and C and N fractions were the main factors affecting the abundance of functional genes that involved in soil N cycling. Our research elucidated a differential response of microbial functional genes to straw mulching and plastic film mulching, which provides a theoretical basis for the further study of soil N cycling mediated by microorganisms in agroecosystems.

## Data availability statement

The datasets presented in this study can be found in online repositories. The names of the repository/repositories and accession number(s) can be found at: https://www.ncbi.nlm.nih.gov/, PRJNA876629.

## Author contributions

JW, FZ, CR, and CY: conceptualization and methodology. YD, MW, NZ, and YL: investigation. YD: writing—original draft preparation. JW and CY: writing—review and editing. All authors contributed to the article and approved the submitted version.

## Funding

This research was funded by the National Natural Science Foundation of China (Grant Nos. 42277322 and 31570440), the Key International Scientific and Technological Cooperation and Exchange Project of Shaanxi Province, China (2020KWZ-010), and the Shaanxi Agricultural Science and Technology Innovation-Driven Project (NYKJ-2021-XA-005 and NYKY-2022-XA-004).

## Conflict of interest

The authors declare that the research was conducted in the absence of any commercial or financial relationships that could be construed as a potential conflict of interest.

The reviewer LF declared a shared affiliation with the author CR to the handling editor at the time of review.

## Publisher’s note

All claims expressed in this article are solely those of the authors and do not necessarily represent those of their affiliated organizations, or those of the publisher, the editors and the reviewers. Any product that may be evaluated in this article, or claim that may be made by its manufacturer, is not guaranteed or endorsed by the publisher.

## Supplementary material

The Supplementary material for this article can be found online at: https://www.frontiersin.org/articles/10.3389/fmicb.2023.1205088/full#supplementary-material

## Footnotes

^1^ https://submit.ncbi.nlm.nih.gov/subs/sra/

^2^https://github.com/OpenGene/fastp, version 0.20.0

^3^ http://cloud.majorbio.com

^4^ https://hku-bal.github.io/megabox/

^5^ http://exon.gatech.edu/GeneMark/metagenome

^6^http://soap.genomics.org.cn/, version 2.21

^7^http://www.diamondsearch.org/index.php/, version 0.8.35

^8^ http://www.genome.jp/kegg
