# Supplementary material for: Effects of straw and plastic film mulching on microbial functional genes involved in soil nitrogen cycling
Source: Front Microbiol. 2023 Jul 11;14:1205088. doi: 10.3389/fmicb.2023.1205088 (PMC10367349; doi:10.3389/fmicb.2023.1205088)

**Supplementary Material**

**Title**: Effects of straw and plastic film mulching on microbial functional genes involved in soil nitrogen cycling

**Authors**: Ying Dou^a^, Mengmeng Wen^a^, Caidi Yang^a^, Fazhu Zhao^a^, Chengjie Ren^b^, Nannan Zhang^a^, Yinyan Liang^a^, Jun Wang ^a, c *^

**Affiliations:**

^a^ Shaanxi Key Laboratory of Earth Surface System and Environmental Carrying Capacity, College of Urban and Environmental Science, Northwest University, Xi’an 710127, China

^b^ College of Agronomy, Northwest A&F University, Yangling 712100, China

^c^ State Key Laboratory of Soil Erosion and Dryland Farming on the Loess Plateau, Institute of Soil and Water Conservation, Chinese Academy of Sciences and Ministry of Water Resources, Yangling, 712100, China

* **Corresponding author**: Jun Wang; E-mail: wangj@nwu.edu.cn

This file includes:

Supplementary Tables S1 to S3; Supplementary Figure S1

**Supplementary Tables**

**Table S1.** Soil basic physicochemical under different mulching. Values are means ± standard error (n = 3). Different letters indicate significant differences (P < 0.05) between the treatments. SM, straw mulching; FM, plastic film mulching; and CK, no mulching. SOC, soil organic C; MBC, microbial biomass C; POC, particulate organic C; PCM, potential C mineralization; STN, soil total N; MBN, microbial biomass N; PNM, potential N mineralization; PON, particulate organic N; NH_4_^+^-N-ammonium N; NO_3_^-^-N-nitrate N.

| Treatment | CK | SM | FM | F | P |
| --- | --- | --- | --- | --- | --- |
| pH | 8.19±0.036a | 8.10±0.031a | 7.97±0.085b | 11.594 | **0.009** |
| SOC(g/kg) | 8.07±0.083b | 8.76±0.400a | 7.43±0.155c | 20.719 | **0.002** |
| MBC (mg/kg) | 309.71±14.461b | 373.52±11.887a | 273.92±13.418c | 43.192 | **<0.001** |
| POC(g/kg) | 1.67±0.08b | 2.7±0.06a | 1.56±0.07b | 70.992 | **<0.001** |
| PCM (mg/kg) | 225.46±3.84b | 289.27±2.82a | 205.25±5.63c | 106.241 | **<0.001** |
| STN(g/kg) | 0.71±0.017ab | 0.79±0.084a | 0.66±0.031b | 4.475 | 0.065 |
| MBN (mg/kg) | 28.6±0.072b | 31.18±0.063a | 27.78±0.091c | 1621.340 | **<0.001** |
| PNM (mg/kg) | 14.87±0.107b | 22.83±0.122a | 14.76±0.099b | 5367.225 | **<0.001** |
| PON(g/kg) | 0.23±0.006b | 1.23±0.012a | 2.23±0.006b | 324.667 | **<0.001** |
| NH_4_^+^-N(mg/kg) | 4.22±0.051c | 6.43±0.075b | 8.31±0.098a | 1720.457 | **<0.001** |
| NO_3_^-^-N(mg/kg) | 41.07±0.524c | 46.25±0.896b | 77.29±0.964a | 1720.457 | **<0.001** |
| C: N | 11.42±0.179a | 11.22±1.428a | 11.27±0.77a | 0.035 | 0.966 |

**Table S2.** The KO number, gene name and detailed functional classification of the investigated genes referring to KEGG database.

| **Classification** | **KO number** | **Name** | **Symbol** |
| --- | --- | --- | --- |
| Ammonification | K01427 | urease | URE |
|  | K01428 | urease subunit alpha | ureC |
|  | K01429 | urease subunit beta | ureB |
|  | K01430 | urease subunit gamma | ureA |
| Nitrification | K10944 | methane/ammonia monooxygenase subunit A | pmoA-amoA |
|  | K10945 | methane/ammonia monooxygenase subunit B | pmoB-amoB |
|  | K10946 | methane/ammonia monooxygenase subunit C | pmoC-amoC |
|  | K10535 | hydroxylamine dehydrogenase | hao |
| Denitrification | K00370 | nitrate reductase / nitrite oxidoreductase, alpha subunit | narG,narZ,nxrA |
|  | K00371 | nitrate reductase / nitrite oxidoreductase, beta subunit | narH,narY,nxrB |
|  | K00374 | nitrate reductase gamma subunit | narI,narV |
|  | K00368 | nitrite reductase (NO-forming) | nirK |
|  | K15864 | nitrite reductase (NO-forming) / hydroxylamine reductase | nirS |
|  | K04561 | nitric oxide reductase subunit B | norB |
|  | K02305 | nitric oxide reductase subunit C | norC |
|  | K00376 | nitrous-oxide reductase | nosZ |
| Dissimilatory nitrate reduction to ammonium | K02567 | nitrate reductase (cytochrome) | napA |
|  | K02568 | nitrate reductase (cytochrome), electron transfer subunit | napB |
|  | K00362 | nitrite reductase (NADH) large subunit | nirB |
|  | K00363 | nitrite reductase (NADH) small subunit | nirD |
|  | K03385 | nitrite reductase (cytochrome c-552) | nrfA |
|  | K15876 | cytochrome c nitrite reductase small subunit | nrfH |
| Assimilatory nitrate reduction | K00367 | ferredoxin-nitrate reductase | narB |
|  | K00372 | assimilatory nitrate reductase catalytic subunit | nasA |
|  | K00360 | assimilatory nitrate reductase electron transfer subunit | nasB |
|  | K00366 | ferredoxin-nitrite reductase | nirA |
|  | K17877 | nitrite reductase (NAD(P)H) | NIT-6 |
| Ammonia assimilation | K00034 | glucose 1-dehydrogenase | gdh |

**Table S3.** Correlation coefficients of Mantel tests between the functional gene composition involved in soil nitrogen cycling and soil properties. Significant (p < 0.05) correlations are shown in bold.

| **Environ-mental attributes** | **Nitrogen** | | **DNRA** | | **ANR** | | **Denitrification** | | **Nitrification** | | **Ammonification** | | **Ammonia assimilation** | |
| --- | --- | --- | --- | --- | --- | --- | --- | --- | --- | --- | --- | --- | --- | --- |
|  | **r** | **p** | **r** | **p** | **r** | **p** | **r** | **p** | **r** | **p** | **r** | **p** | **r** | **p** |
| pH | **0.016** | 0.411 | -0.092 | 0.565 | 0.107 | 0.235 | 0.294 | 0.138 | -0.174 | 0.67 | **0.535** | **0.027** | 0.070 | 0.369 |
| SOC | 0.474 | **0.020** | **0.760** | **0.001** | -0.172 | 0.794 | -0.251 | 0.885 | 0.032 | 0.374 | **0.672** | **0.004** | -0.063 | 0.541 |
| POC | 0.638 | **0.003** | **0.695** | **0.004** | -0.030 | 0.514 | -0.142 | 0.78 | 0.051 | 0.358 | 0.247 | 0.077 | -0.108 | 0.718 |
| PCM | 0.638 | **0.003** | **0.655** | **0.002** | -0.050 | 0.584 | -0.178 | 0.864 | -0.007 | 0.506 | **0.470** | **0.012** | 0.031 | 0.307 |
| MBC | 0.627 | **0.006** | **0.497** | **0.005** | -0.084 | 0.668 | -0.164 | 0.822 | 0.151 | 0.207 | **0.455** | **0.017** | 0.134 | 0.157 |
| STN | 0.220 | 0.263 | 0.145 | 0.167 | -0.234 | 0.897 | 0.061 | 0.334 | 0.405 | 0.156 | 0.180 | 0.255 | 0.182 | 0.266 |
| PON | 0.616 | **0.007** | **0.625** | **0.005** | 0.040 | 0.366 | -0.118 | 0.696 | -0.010 | 0.485 | 0.254 | 0.078 | -0.060 | 0.556 |
| PNM | 0.645 | **0.007** | **0.651** | **0.009** | 0.022 | 0.419 | -0.113 | 0.707 | 0.033 | 0.433 | 0.205 | 0.077 | -0.059 | 0.555 |
| MBN | 0.601 | **0.007** | **0.628** | **0.004** | 0.002 | 0.438 | -0.091 | 0.676 | 0.065 | 0.333 | **0.412** | **0.02** | -0.032 | 0.474 |
| NH_4_^+^-N | **-0.047** | 0.560 | 0.013 | 0.457 | 0.091 | 0.228 | 0.271 | 0.058 | 0.000 | 0.505 | **0.345** | **0.036** | 0.032 | 0.319 |
| NO_3_^-^-N | -0.068 | 0.597 | 0.025 | 0.389 | -0.100 | 0.711 | 0.030 | 0.376 | 0.170 | 0.183 | **0.684** | **0.015** | 0.084 | 0.204 |
| C: N | 0.155 | 0.292 | 0.189 | 0.177 | -0.286 | 0.958 | -0.138 | 0.716 | 0.247 | 0.202 | 0.074 | 0.358 | 0.187 | 0.215 |

**Figure S1** Taxonomic assignment of typical functional genes coding for: (a) ureC; (b) ureA;(c) nirB;(d) nirD; (e) napA; (f) nrfA at the genera level. SM, straw mulching; FM, plastic film mulching; and CK, no mulching.


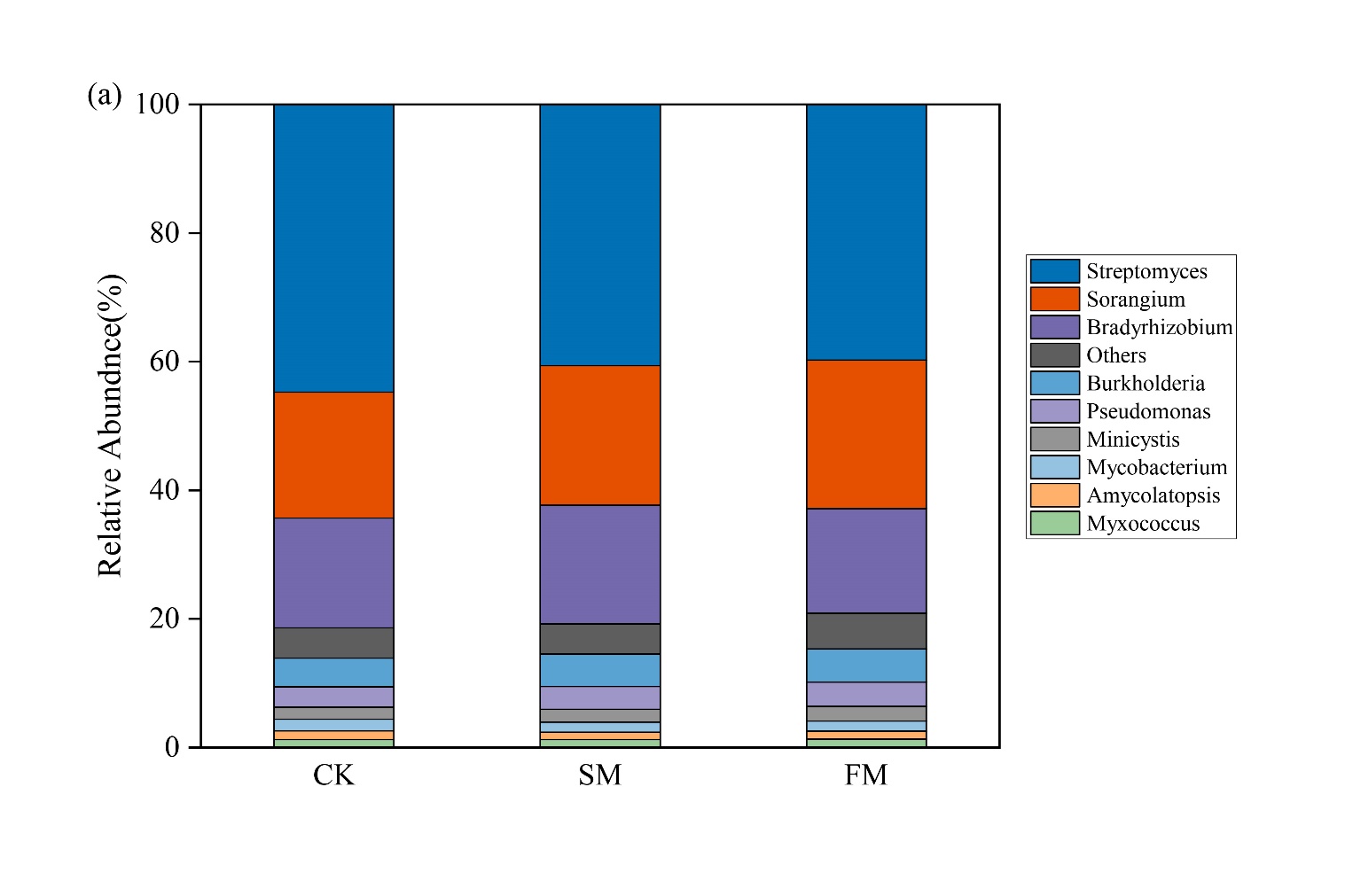


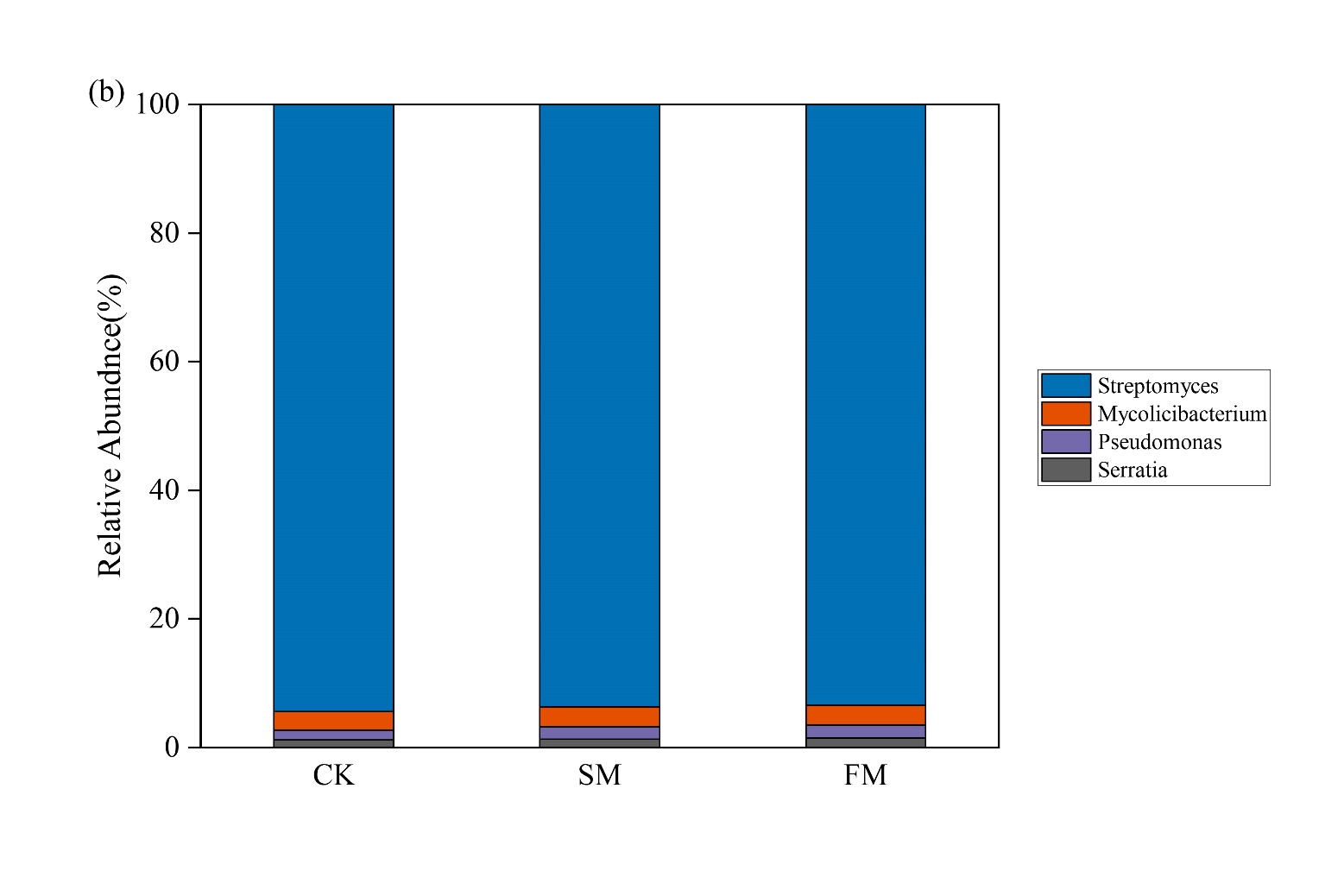


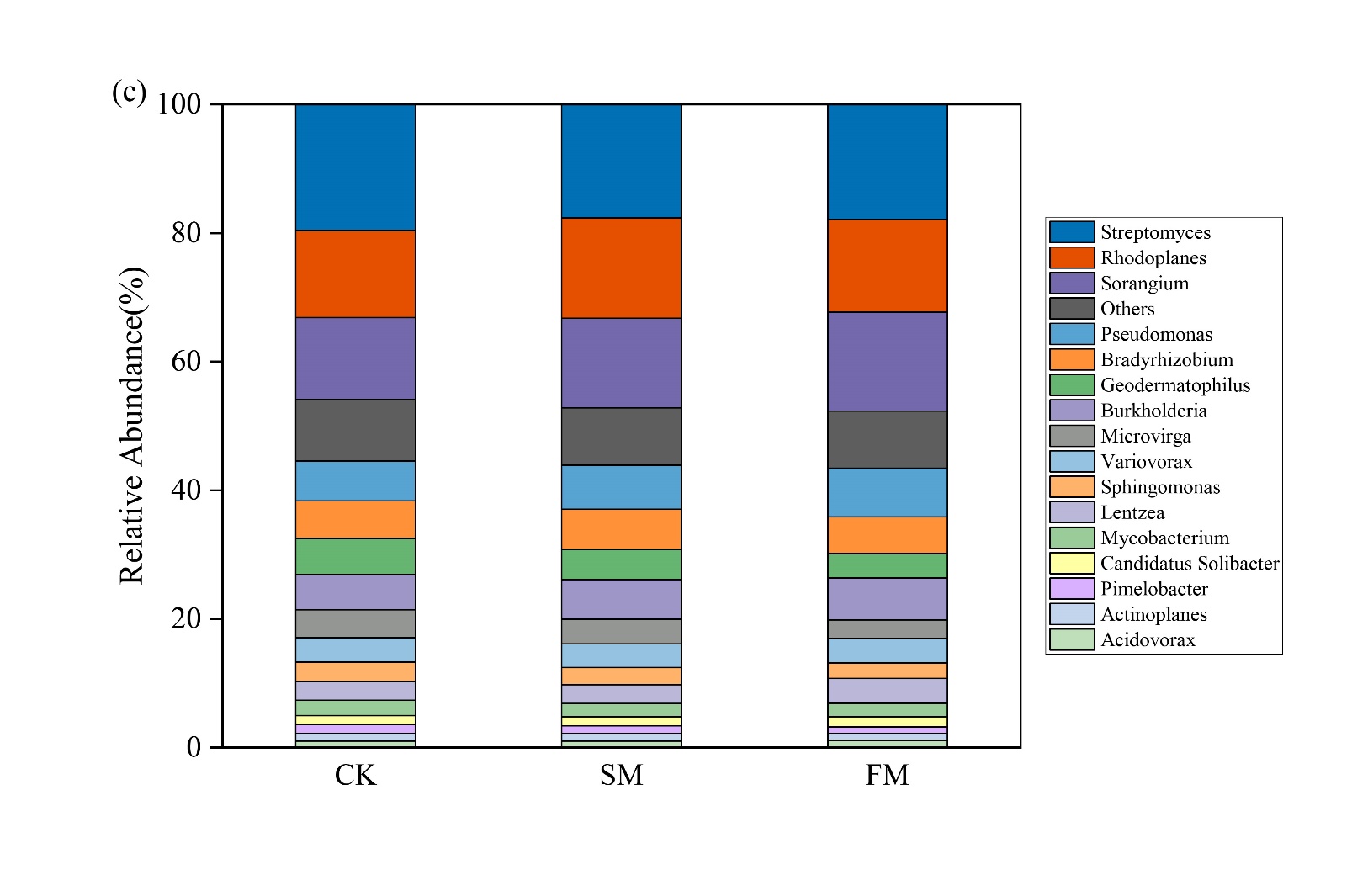

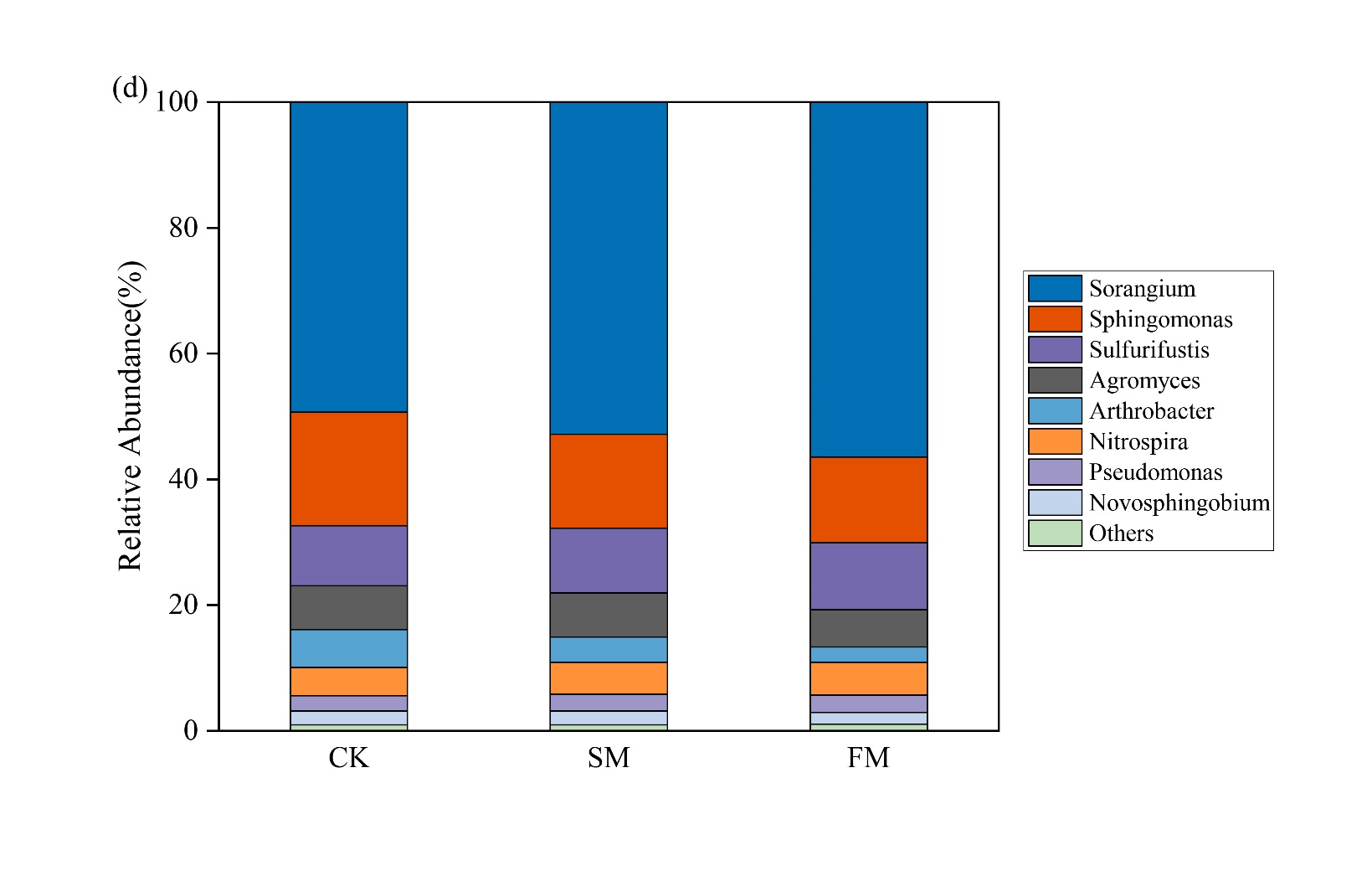


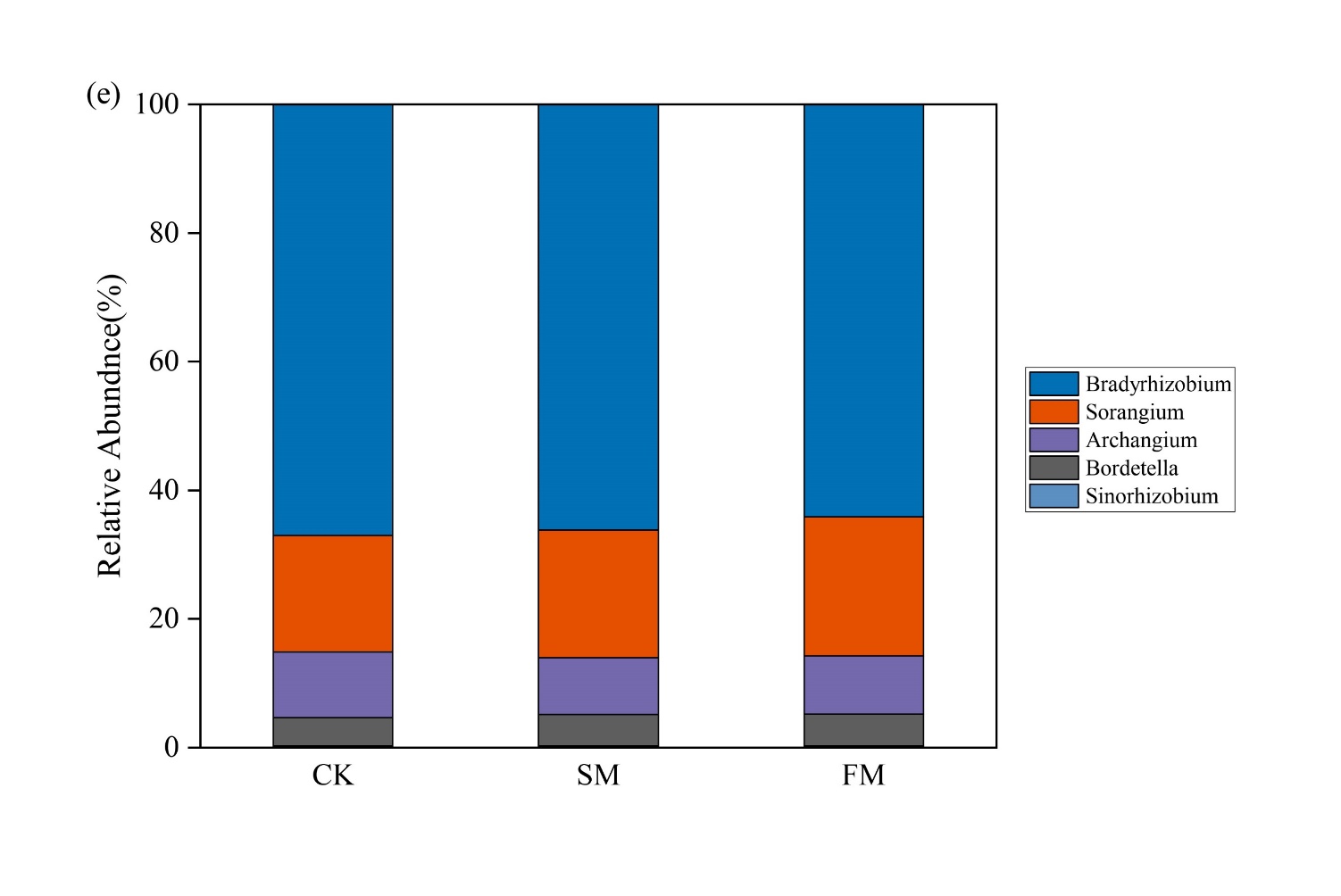


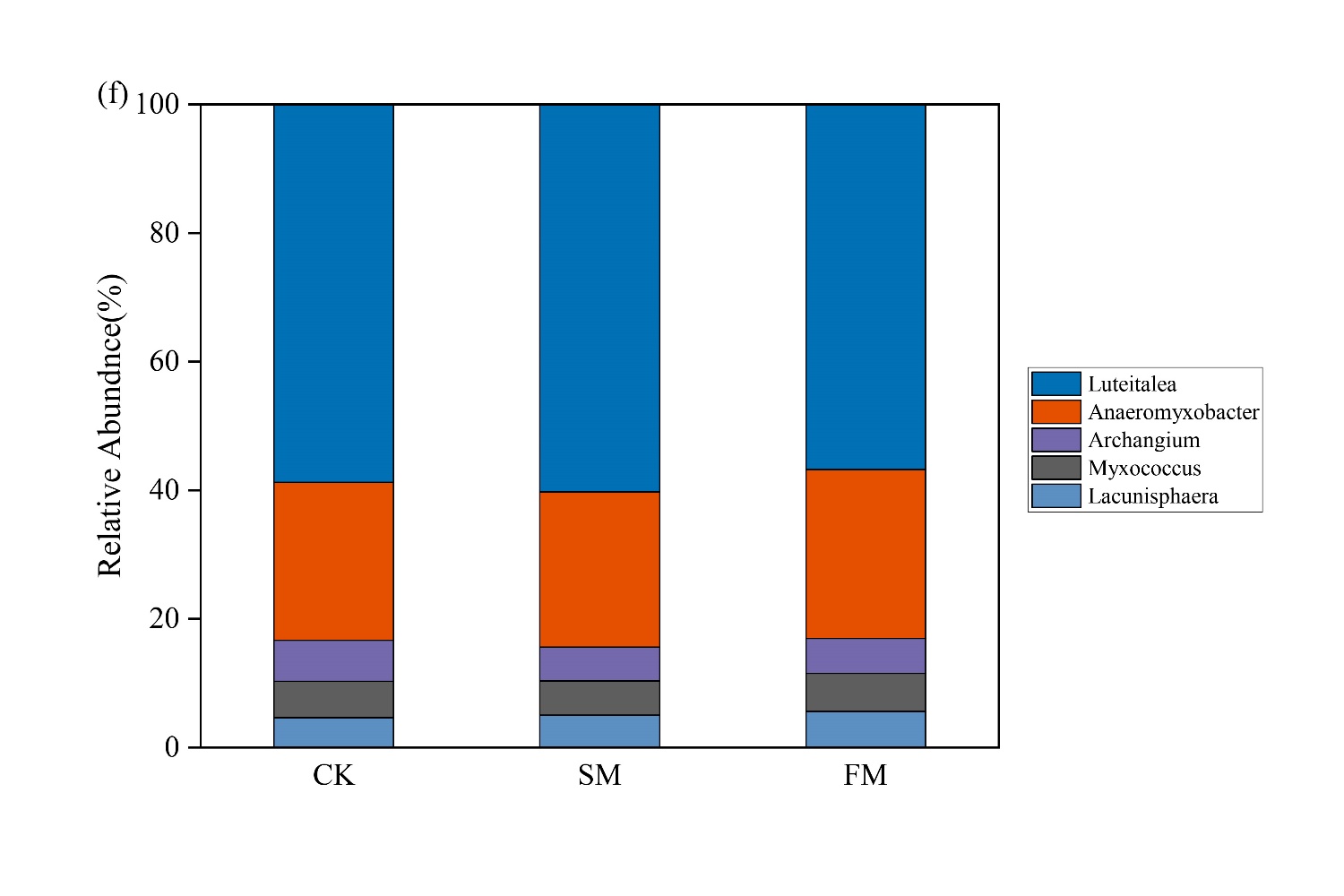

Supplement: Supplementary file 1 [file Data_Sheet_1.docx]
